# Familial hepatocellular carcinoma in an endemic area: two case reports

**DOI:** 10.1186/s13104-015-1366-7

**Published:** 2015-09-05

**Authors:** Elroy P. Weledji, Dickson S. Nsagha, George Enoworock, Maurice Mouladje

**Affiliations:** Department of Surgery, Faculty of Health Sciences, University of Buea, PO Box 126, Limbe, S.W. Region, Buea, Cameroon; Department of Public Health, Faculty of Health Sciences, University of Buea, Buea, Cameroon; Department of Pathology, Regional Hospital Buea, Buea, Cameroon

**Keywords:** Hepatocellular carcinoma, Fibrolamellar variant, Hepatitis B, Genetics

## Abstract

**Background:**

Hepatocellular carcinoma (HCC) usually affects patients aged 50–70 years but earlier onset (25–40 years) may occur in hepatitis B endemic areas. 70–90 % of HCC develop on a background of cirrhosis. However, hepatitis B virus is directly oncogenic and can cause HCC in the absence of cirrhosis. This may represent a major cause of death from late diagnosis in resource-limited areas.

**Case presentation:**

We report a black African family in which clinical diagnosis of HCC was made on two male siblings in the south west region of Cameroon.

**Conclusions:**

The highest risk for HCC may occur in families in which a hereditary component may be acting in concert with hepatitis B virus. In all cases of HCC, it is important to screen all first degree relatives to detect early and asymptomatic disease.

**Electronic supplementary material:**

The online version of this article (doi:10.1186/s13104-015-1366-7) contains supplementary material, which is available to authorized users.

## Background

Hepatocellular carcinoma (HCC) is the fifth most common cancer worldwide, with more than 80 % of cases found in endemic areas of hepatitis B such as Africa or East Asia. The rising incidence in the West is due to increasing incidence of hepatitis C infection [1]. 70–90 % of HCC develop on background of cirrhosis. Hepatitis B is strongly associated (100 fold increase in risk) and alcoholic cirrhosis is the commonest predisposing factor in the Western world. HCC occurs from the processes of chronic injury and regeneration (cirrhosis), and dysplasia. Thus it usually affects patients aged 50–70 years [2]. However, hepatitis B virus (HBV) is directly oncogenic by incorporating into host genetic material and can cause HCC in the absence of cirrhosis. Thus an earlier onset (25–40 years) may be seen in endemic areas with earlier exposure [2–4]. The rare fibrolamellar variant in non-cirrhotic livers of young adults is a distinct entity not associated with hepatitis B infection. It is due to a heterozygous deletion on chromosome 19 that encodes a functional chimeric protein (DNAJB1-PRKACA) and characterized by being less aggressive with a normal alpha-fetoprotein (AFP) tumour marker and a female predominance [5, 6]. This is in contrast to conventional HCC which has a twofold to fourfold male predominance and occurs mostly in hepatitis B endemic areas [5]. Chronic hepatitis, cirrhosis of the liver, and HCC are known to be related to the hepatitis B surface antigen (HBsAg) positive rate suggesting the possibility of maternal transmission [4, 7]. The genetic abnormality that lead to HCC are not yet known but new genetic markers for diagnosis of hepatitis C related HCC have been demonstrated [8]. Only limited attention has been given to the role of primary genetic factors in HCC, but scattered anecdotal reports have identified familial aggregations of HCC (Table 1) [7]. Being rare familial fibrolamellar HCC has never been reported [6]. We propose that greater attention should be given to the role of primary genetic factors in HCC and that appropriate consideration be given to interaction with environmental factors, such as HBV infection and aflatoxin exposures [6, 9].

**Table 1:** Familial hepatocellular carcinoma

Kaplan and Cole [20]	3 male adult siblings aged 64, 49 and 44
Hagstrom and Baker [21]	3 male siblings aged 11, 22 and 31
Denison et al. [22]	Familial hepatoma on background micronodular cirrhosis
Ohbayashi et al. [17]	Familial clustering of asymptomatic carriers
Gilmore et al. [29]	3 or 4 male siblings in Chinese family
Lynch et al. [7]	2 familial aggregations in Costa Rica
Harvey et al. [23]	3 male siblings aged 33, 43, 46
Chang et al. [24]	2 pairs of young brothers (5 and 7years) and (9 and 7years)
Lok et al. [25]	Morbidity and mortality from chronic hepatitis B virus infection in family members
Alberts et al. [26]	Clustering of hepatocellular carcinoma in Alaska Native families
Weledji et al. [3] *BMC Research notes*	2 male siblings aged, 24 and 35 in endemic area in Cameroon

## Case presentation

**Fig. 1:**
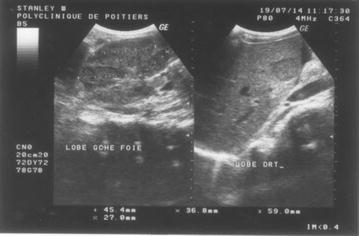
Transabdominal ultrasonography of liver showing multinodular HCC in left lobe and a nodular HCC with posterior enhancement in right lobe

A 35-year- old black African male presented with a 3-month history of fever, and vague upper abdominal pain and progressive abdominal distension. This was associated with rapid weight loss and anorexia. He also complained of difficulty in breathing because of the abdominal distension and could only sleep upright whilst sitting on a chair. He had no altered bowel habit and the stools and urine were of normal colour. There was no past history of jaundice or hepatitis and he did not smoke nor consumed alcohol. He had his normal childhood vaccinations. The patient had been otherwise healthy and working for an airline hotel company. His younger brother had died 9 years previously, aged 24, from liver cancer. He had undergone an ‘open and close’ laparotomy for a large HCC on the left lobe. His symptoms and signs were almost the same as his sibling. He had moderate ascites but no stigmata of chronic liver disease. He suffered agonizing pain in the terminal stage of the illness for which I palliated with narcotics. On examination of the senior brother 9 years after, apart from the respiratory distress the vital signs were within normal limits. He was not jaundice, but cachetic with gross ascites. There was severe bilateral pitting oedema of the legs with no palpable lymphadenopathy. There was a palpable non-tender nodular liver in the epigastrium but no peripheral stigmata of chronic liver disease such as leuconychia, palmar erythema, spider naevi, koilonychia, clubbing, jaundice, splenomegaly etc. An Upper gastrointestinal endoscopy in the early stage of his illness had revealed a healed antral ulcer but no oesophageal varices. An ultrasound scan confirmed ascites and a heterogenous mass of 9 cm in the left lobe of the liver. There were also few nodules in the right lobe with greatest diameter of 4 cm. There was no paraortic lymphadenopathy nor splenomegaly. The picture was consistent with HCC of a nodular type (Fig. 1). A chest X-ray was normal. A hepatitis screen for HBsAg and hepatitis C virus antibody (HCV Ab), along with human immunodeficiency virus (HIV) serology were negative. His haemoglobin level was normal (14 g/dl). Liver function tests and serum biochemistry were not available but serum alpha fetoprotein was within the normal limits (1.14; normal range <8.5 ng/ml). A diagnostic ascitic tap demonstrated a yellowish exudate (protein 50.3 g/lg/l) with predominantly lymphocytes consistent with malignancy or inflammation. He was put on a salt-free diet and commenced on spironolactone 100 mg/day for 5 days which was increased to 200 mg/day thereafter. The malignant ascites remained refractory and a therapeutic paracentesis, with intravenous colloid replacement removed 3 l ascitic fluid per day for 3 days. His respiratory distress subsided and pitting oedema diminished. Just as we planned to do a percutaneous biopsy of the liver lesion, he deteriorated rapidly, with generalized weakness that required assistance with his oral feeding. He died a few days after.

## Conclusions

With respect to the natural history of hepatitis B, it takes 10 years to develop chronic hepatitis, 20 years to develop cirrhosis and 30 years to develop HCC [3, 4]. The rapid development of ascites, increasing liver size or jaundice, in a patient with known cirrhosis would always suggest HCC [4]. Although there was no histological verification, a clinical diagnosis of early-onset HCC was made in the two male siblings based on the fever, weight loss, right hypochondrial pain from nodular liver lesions and refractory ascites. There was no evidence of portal hypertension as there was no stigmata of chronic liver disease, the ascites was exudative and the ultrasonic examination did not reveal an underlying cirrhosis [10]. The bilateral limb oedema was due to hypoalbuminaemia more from nutritional insufficiency than from liver dysfunction. The differential diagnosis would include the rare fibrolamellar variant of HCC (FHCC) especially as the hepatitis screen was negative and it typically affects adolescents and young adults with often no coexistent liver disease nor history of hepatitis B infection [8]. Although FHCC mostly occurs in non-hepatitis B endemic areas, some cases have been reported in relatively endemic areas [11]. It is characterized by the lack of symptoms until the tumour is sizeable and is thus often advanced when diagnosed as in these siblings. Serum neurotensin, a new tumour marker for FHCC may discriminate it from HCC especially as a negative or normal value of alpha feto-protein tumour marker does not exclude an HCC [6]. Alpha feto-protein tumour marker is elevated in only 50–60 % of cases of HCC [12]. Global gene expression profiling revealed a small set of genes, SPINK 1, a secretory trypsin inhibitor as a potential HCC marker [13]. Although operator dependent, ultrasound scan is capable of detecting 2 cm lesions, but further characterization by computed tomography (CT) or magnetic resonance imaging (MRI) is necessary [14]. Biopsy is usually not indicated except in specialist centres, or in inoperable cases. It is considered to carry a risk (1–2 %) of tumour seeding along the needle track, which may convert an operable tumour into an inoperable one [15]. A CT scan with or without biopsy of non-tumour liver is useful if in doubt of an underlying liver disease. CT chest ± bone scintigraphy would also exclude extrahepatic disease [3]. Tumour differentiation and vascular invasion are important predictors of survival after surgical resection or liver transplantation for HCC [16].

There must be a genetic basis for the aggressive liver malignancy occurring in two siblings during their early adulthood. It is unlikely to be by chance. The early age of onset especially in the 24-year old, harbour important implication for hereditary susceptibility to this lesion [6, 17]. Being in an endemic area it is very likely that the brothers were carriers of HBsAg. Even though the serology was negative, occasionally infection without detectable serum HBsAg occurs. In these cases hepatitis B virus DNA (HBVDNA) and hepatitis B core antibody (HBcAb) tests would be mandatory [18]. In addition changes in HBsAg variants in carrier children before and after universal vaccination have been reported [19]. Up to 20 % of the population in these highly endemic areas may be asymptomatic HBsAg positive chronic carriers [1, 4]. Vertical transmission of the hepatitis B virus is probably a major reason for the high incidence of hepatitis B infection in the endemic areas, infants acquiring the virus from infected mothers in utero, at birth or shortly after birth (Fig. 1) [4]. With the impaired immunity of the baby compared with later life the infant is then at increased risk of developing persistent hepatitis B carriage, chronic active hepatitis, progressive liver damage and hepatocellular cancer [4]. The reason for some individuals becoming chronic HBsAg carriers, with development of chronic liver disease or HCC in their adult years remains unclear. Genetic contribution may thus explain the presence of healthy or non-healthy carriers of HBsAg [4, 6]. The earlier onset of HCC in the younger sibling may indicate a greater genetic contribution to persistent infection than in the older sibling. Familial occurrence of HCC was first reported by Kaplan and Cole which involved 3 male adult siblings aged 64, 49 and 44 [20]. Hagstrom and Baker described HCC in 3 male siblings aged 11, 22 and 31 and with no evidence of associated hepatic disease [21]. Denison et al. reported familial hepatoma on a background micronodular cirrhosis associated with hepatitis-associated antigen [22]. Lynch et al. reported two familial aggregations of HCC from Costs Rica [7]. The maternal transmission of HBsAg and susceptibility to HCC as suggested by the pattern of involvement, with the mother and three sons (one of a different father) affected was demonstrated by Harvey et al. [23]. Chang et al. reported fraternal hepatocellular carcinoma in young children in two families [24]. Alberts et al. reported clustering of hepatocellular carcinoma in Alaska native families [25]. Lok et al. Revealed the morbidity and mortality from chronic hepatitis B virus infection in family members of patients with malignant and non-malignant hepatitis B virus—related chronic liver disease [26]. Gilmore et al. observed familial clustering of HCC in a Chinese family and concluded that HCC observed in 3 or 4 male siblings even in the presence of hepatitis B infection seemed statistically unlikely to occur by chance [27]. It was therefore suggested that another risk factor such as environmental (aflatoxin from fermented crops) exposure in certain parts of Africa and/or genetic factors may be involved [8, 28, 29]. Being rare, familial fibrolamellar hepatocellular carcinoma clustering has never been reported [7, 30, 31]. HCC-prone families of the type reported here could provide powerful models for studying the preventive measures of a hepatitis B vaccination [32, 33]. It would seem prudent that hepatitis vaccination be given the highest priority to those individuals where the HCC yield is increased. The optimum timing for immunization in conjunction with the administration of hepatitis B immunoglobulin at a contralateral site is immediately after birth or within 12 h [34]. We suggest a more extensive investigation of the genetic hypothesis of HCC and its variant (FHCC).

**Fig. 2:**
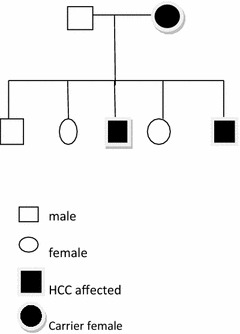
Family tree of two generations depicting carrier state commoner in males

Although not histologically verified because of the refractory ascites and minimal resources including autopsy, our observations document familial clustering of HCC. The highest risk for HCC may occur in families in which a hereditary component may be acting in concert with hepatitis B virus. In all cases of HCC it is therefore important to screen for genetic markers of HCC in all first degree family members and selected second degree relatives so as to detect early and asymptomatic disease (Fig. 2).

## Consent

“Written informed consent was obtained from the parents of the deceased for publication of this case report and any accompanying images. A copy of the written consent is available for review by the Editor-in-Chief of this journal.”

## Additional file

**Additional file 1.** Familial hepatocellular carcinoma.

## Abbreviations

HCC: hepatocellular carcinoma
FHCC: fibrolamellar hepatocellular carcinoma
HepBsAg: hepatitis B surface antigen
HCV Ab: hepatitis C antibody
HBDNA: hepatitis B DNA
HBcAb: hepatitis B core antibody
